# Closing the Gap

**Published:** 1924-05

**Authors:** 


THE
### HOSPITAL and HEALTH
REVIEW

### CLOSING THE GAP.

So far as London is concerned the annual meeting of the President and General Council of King Edward's Hospital Fund is the most outstanding event in the hospital year. To it are presented in authoritative form summaries of the financial and other progress of the preceding twelve months, and abundant opportunity is provided for drawing inferences and deductions as to the prospects of the immediate future. Those conclusions are, happily, once more increasingly favourable. The gap between income and expenditure is steadily growing narrower. In 1922 it was less than half the width which we had to deplore in 1920, and although the exact figures for last year are not ascertained they are known to be so substantially less than the £174,000 of 1922 that we may take it that the gap is very nearly closed. Thanks to benefactions, some hospitals actually had a surplus, but, gratifying as that result must be, we have to look upon the London hospitals as a whole, and so long as any of them have balances seriously on the wrong side, the position will continue to give ground for anxiety. Last year King Edward's Fund did well. It distributed £235,000, which was £15,000 more than in 1922. Looking at the financial position of the country this is an excellent result, and as trade improves and unemployment diminishes we may fairly expect even more encouraging figures. The hospitals, both in the capital and the provinces, must ever depend upon the steady and increasing flow of systematic contributions—benefactions by will are a luxury, welcome enough when they come, but not to be counted upon by any prudent and conservative system of hospital finance. It is, therefore, gratifying to know that there was last year an increase in the annual subscriptions to King Edward's Fund.

The experience of London is, broadly speaking, that of the great towns, with one or two conspicuous and unhappy exceptions. Notwithstanding the difficulties experienced by most people in consequence of heavy taxation and lessened incomes, voluntary contributions are increasing, and payments from patients are assuming an ever-growing importance. There is no longer any emergency fund to temper the wind to the shorn lamb, nor, to be frank, is it desirable that there should be. The State has helped the hospitals over the stile, but it is of paramount importance that they should remain distinctly and essentially voluntary institutions, ever dependent upon the good will and the sense of duty of the country at large. That good will has not been lacking, and the result does credit both to the people and the hospitals. The cost of working any hospital is now something like two and a-half times what it was before the war—an increase which might well make the heart of the stoutest optimist quail. But a well-informed and cautious optimism is always an enormous asset, and the hospitals enjoy their full share of it. The men who are chiefly responsible for hospital policy are cheerful men who have seen so many acute difficulties surmounted that they are not appalled by fresh ones. Moreover, the serious shock which the war gave to the voluntary principle has at once sharpened the instinct towards economy and further awakened the latent ingenuity which devises new and fruitful sources of revenue. The mass-contribution system, for instance, which was in existence before the war, has been enormously extended with admirable results. Yet it is only in its infancy, and will not reach its full development until it is working in every factory, shop, and office in the country. It is for the masses to save and maintain the hospitals as voluntary institutions, and no praise is too high for the manner in which the working classes during these after-war years have recognised and fulfilled their responsibility.

But although the future is infinitely brighter than it was two or three years ago, it is by no means unclouded. Unless there should be a substantial fall in the cost of all the necessaries of life—and at present the fall is very small and very slow—the maintenance of hospitals will continue to be costly. Indeed, we can only expect that any saving in that respect will be more or less neutralised by the increased cost of scientific equipment. Hospitals are no longer merely places where disease is cured or alleviated. They are laboratories, humming with chemists and experimenters seeking how to prevent disease, and full of students who find it difficult to absorb and co-ordinate the new knowledge. These preventive activities are costly and need space as well as men. Moreover, the pressure upon beds is ever growing. Waiting lists of hundreds are common enough; in a few cases they are growing into the thousands. All this means modernisations and extensions, more space for patients and nurses and scientific staffs. It is probably not an exaggeration to say that one-half of the hospitals in the country are in need of enlargement or removal, while additional ones are demanded in no small number. During the next few years vast sums will have to be spent upon those bricks and mortar which have become the rich man's luxury. If they are not so spent the hospitals, which are repidly becoming the refuge in illness of all classes of the community, will fail to perform their functions, and will inevitably have to face a renewed demand for their nationalisation. Voluntary effort, we shall be told, has finally failed, and we must henceforth look to the State to provide hospital treatment for every one who chooses to demand it. This would be a moral disaster from which the national character would suffer in grievous measure. The voluntary system means self-respect; hospitals maintained out of taxes would mean something not readily distinguished from pauperisation.

